# Low-Threshold and Long-Term Stable Soliton Fiber Laser Using PbSe/PbS Quantum Dot-Polystyrene Composite Saturable Absorber

**DOI:** 10.3390/nano16050306

**Published:** 2026-02-27

**Authors:** Bin Yang, Jingping Shao, Chunxiao Liu, Ling Yun, Zuxing Zhang

**Affiliations:** Advanced Photonic Technology Lab, College of Electronic and Optical Engineering & College of Flexible Electronics, Nanjing University of Posts and Telecommunications, Nanjing 210023, China; b22021505@njupt.edu.cn (B.Y.); 1224025101@njupt.edu.cn (J.S.); chunxiaoliu@njupt.edu.cn (C.L.); zxzhang@njupt.edu.cn (Z.Z.)

**Keywords:** PbSe/PbS quantum dots, PbS overcoating, polymer composite film, saturable absorber, bidirectional pumping, mode-locking

## Abstract

Colloidal PbSe quantum dots are promising candidates as saturable absorbers for ultrafast fiber lasers, but their performance is often limited by surface-related defects and chemical instability, leading to aggregation under optical pumping. In this study, we present a freestanding PbSe/PbS quantum dot-polystyrene composite saturable absorber film, with PbS overcoating on PbSe to enhance surface passivation and oxidation resistance. The composite exhibits a saturation intensity of 5.76 kW·cm^−2^, a modulation depth of 33%, and an optical damage threshold of 13.6 mJ·cm^−2^. When integrated into a bidirectionally pumped erbium-doped fiber laser in the anomalous-dispersion regime, the device demonstrates self-starting soliton mode locking at an ultralow pump threshold of 6 mW, generating 1.06 ps pulses with a radio-frequency signal-to-noise ratio of approximately 65 dB. The spectra remain stable over an 8-month period, showing excellent environmental and operational durability. These findings confirm that PbSe/PbS quantum dots in a polymer matrix offer a robust, low-threshold saturable absorber platform for ultrafast fiber lasers.

## 1. Introduction

Ultrafast fiber lasers deliver femtosecond-to-picosecond pulses and support a wide range of high-precision photonic tasks, including material processing, nonlinear diagnostics, and biophotonic measurements [1,2,3]. Among various techniques, passive mode locking remains the most practical route to stable ultrashort pulse generation, where the saturable absorber (SA) governs pulse initiation and sustains steady pulse evolution in the cavity [4,5,6]. In recent years, nanomaterial SAs—especially quantum dots (QDs)—have attracted extensive interest because they offer bandgap tunability, intrinsically fast carrier dynamics, and compact fiber-compatible packaging [7,8,9,10]. Despite these merits, many colloidal QDs (e.g., PbSe and PbS) possess reactive surfaces and defect-associated trap states [11]. Under continuous optical pumping, such surface-related effects can accelerate oxidation and induce aggregation, which weakens the nonlinear absorption response and undermines long-term operational stability, thereby limiting their use in reliable ultrafast laser platforms.

Inorganic surface passivation via overcoating has therefore been explored to mitigate surface-driven degradation, leading to overcoated or heterostructured QD SAs [12,13,14]. Introducing an overcoating layer can reduce trap-assisted loss, slow chemical deterioration, and improve optical robustness, which is beneficial for stable saturable absorption. Nevertheless, even for heterostructured QD systems such as PbS/CdS and InP/ZnSeS/ZnS, practical challenges remain in power handling, environmental durability, and self-starting operation at low pump thresholds [15,16]. These considerations motivate the development of QD-based SAs that can simultaneously provide low saturation intensity, high power tolerance, and long-duration stability in fiber laser cavities.

PbSe/PbS represents a favorable material combination for improving QD-SA durability. PbSe and PbS share the rock salt lattice and exhibit minimal mismatch, which facilitates PbS overcoating on PbSe and helps suppress defect formation at the PbSe/PbS interface [17,18]. In addition, the PbS overcoating can act as a protective barrier against oxygen and moisture, while its thermal characteristics may assist heat dissipation under intense intracavity irradiation [19,20,21]. Combined with the broad bandgap tunability and large exciton Bohr radius of PbSe [22], PbS-overcoated PbSe (denoted as PbSe/PbS) QDs are promising for achieving effective saturable absorption with enhanced operational stability [23,24]. Building on earlier QD-SA studies [25], we adopt a PbSe/PbS design and a freestanding QD-polystyrene (PS) composite film enabling connector-level integration, aiming to achieve a low self-starting threshold and improved long-term stability.

Here, we implement this freestanding PbSe/PbS QD-PS composite SA in a bidirectionally pumped erbium-doped fiber laser operating in the anomalous-dispersion regime. The composite film exhibits a saturation intensity of 5.76 kW·cm^−2^, a modulation depth of 33%, and an optical damage threshold of 13.6 mJ·cm^−2^. Using this device, self-starting soliton mode locking is realized at a total pump threshold of 6 mW, producing 1.06 ps pulses with a radio-frequency signal-to-noise ratio (SNR) of ~65 dB. Moreover, the output spectra show no observable degradation over 8 months of monitoring.

## 2. Synthesis and Characterization

**Figure 1:**
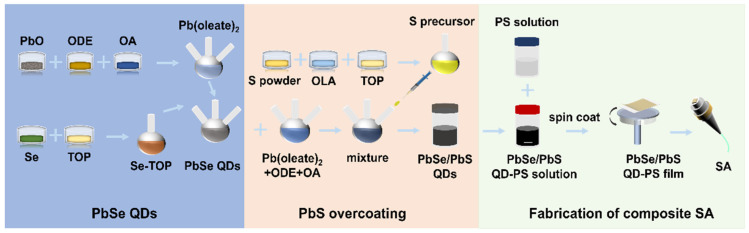
Schematic illustration of PbSe QD synthesis, PbS overcoating to obtain PbSe/PbS QDs, and fabrication of the freestanding PbSe/PbS QD-PS composite SA.

The preparation of PbSe QDs, subsequent PbS overcoating to obtain PbSe/PbS QDs, and fabrication of the freestanding QD-PS composite SA film are summarized in Figure 1. PbO (1 mmol, 90%, Sinopharm, Shanghai, China) was first dehydrated under vacuum at 120 °C for 20 min. After adding 1-octadecene (20 mL, ODE, 90%, Sigma-Aldrich, St. Louis, MO, USA) and oleic acid (2 mmol, OA, 90%, Alfa Aesar, Ward Hill, MA, USA), the mixture was maintained at 110 °C for 2 h under nitrogen to form Pb(oleate)_2_. In parallel, Se powder (1 mmol, 99.5%, Alfa Aesar, Ward Hill, MA, USA) was dissolved in trioctylphosphine (5 mL, TOP, 90%, Sinopharm, Shanghai, China) and heated at 120 °C for 15 min to yield the Se-TOP precursor. The Se-TOP solution was then injected rapidly into the Pb(oleate)_2_ solution with vigorous stirring. After a 5-min growth period, the reaction was quenched in an ice bath. The obtained PbSe QDs were purified by centrifugation (10,000 rpm, 10 min) and redispersed in 10 mL ODE containing 1 mmol OA.

PbS overcoating was performed by adding Pb(oleate)_2_ (0.25 mmol), ODE (5 mL), and OA (0.5 mmol) to the purified PbSe dispersion and holding the mixture at 120 °C. The sulfur precursor was prepared by dissolving S powder (0.2 mmol, 99.5%, Alfa Aesar, Ward Hill, MA, USA) in oleylamine (2 mL, OLA, 90%, Sigma-Aldrich, St. Louis, MO, USA) and then mixing with TOP (2.5 mL). The mixture was ultrasonicated until complete dissolution. This sulfur precursor was injected at 2 mL·min^−1^, allowed to react for 5 min, and then the solution was rapidly cooled to room temperature. The resulting PbSe/PbS QDs were further purified by centrifugation.

To fabricate the freestanding SA film, PS (1 g, Macklin, Shanghai, China) was dissolved in toluene (10 mL, Sinopharm, Shanghai, China) and ultrasonicated (40 kHz, 15 min) to obtain a 100 mg·mL^−1^ PS solution. The PbSe/PbS QD dispersion was mixed with the PS solution at a 1:1 volume ratio and sonicated (100 W, 20 min) to form a uniform QD-PS composite ink. The ink was spin-coated onto a quartz substrate; after solvent evaporation, the film was peeled off and transferred onto the end face of an FC/PC connector, forming an all-fiber SA device.

**Figure 2:**
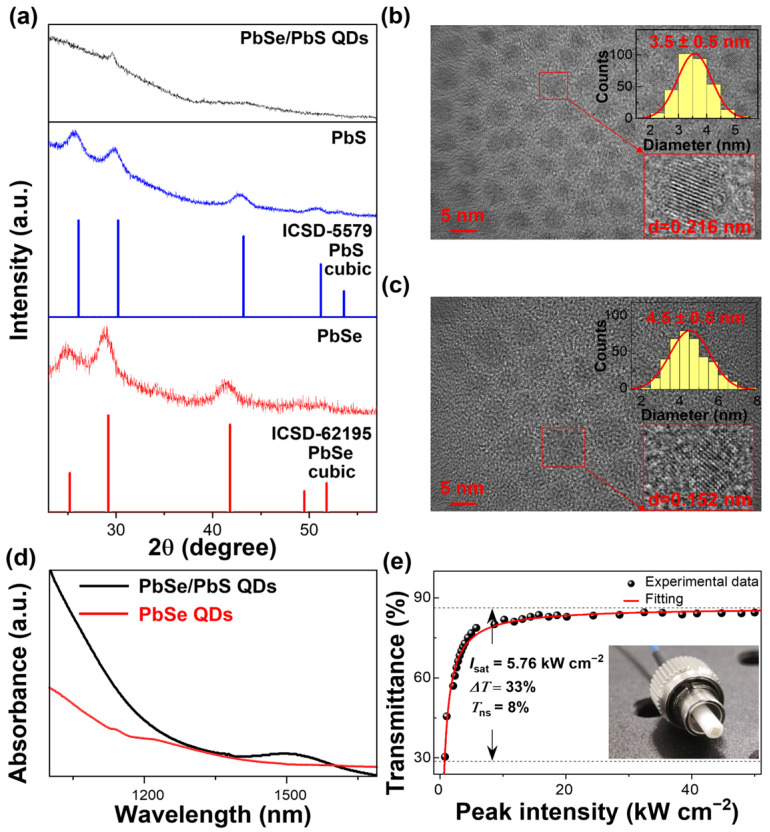
(**a**) XRD patterns of PbSe, PbS, and PbSe/PbS QDs. (**b**) TEM image of PbSe QDs; insets: size distribution histogram (top) and HR-TEM image (bottom). (**c**) TEM image of PbSe/PbS QDs; insets: size distribution histogram (top) and HR-TEM image (bottom). (**d**) Absorption spectra of PbSe and PbSe/PbS QDs. (**e**) Nonlinear transmission of the PbSe/PbS QD-PS composite SA film; inset: photograph of the freestanding composite film/SA device.

Figure 2a compares the X-ray diffraction (XRD) patterns of PbSe, PbS, and PbSe/PbS QDs. Relative to the individual PbSe and PbS samples, the PbSe/PbS QDs exhibit broader diffraction peaks (larger FWHM). Such peak broadening can arise from the combined effects of reduced coherence length (size/structural disorder) and/or microstrain introduced during the PbS overcoating process [26,27]. This observation is consistent with structural modification and interfacial lattice accommodation when PbS is introduced onto the PbSe QD surface to form PbSe/PbS QDs [18]. Transmission electron microscopy (TEM) images (Figure 2b,c) show nearly spherical nanoparticles with narrow size distributions. The mean diameter increases from 3.5 ± 0.5 nm (PbSe) to 4.5 ± 0.5 nm (PbSe/PbS), consistent with the formation of a thin PbS overcoating layer. Under the simplifying assumption of isotropic radial growth, this average size increase corresponds to an effective overcoating thickness of ~0.5 nm. We note that this estimate is approximate because overcoating may be nonuniform and the size statistics are limited by TEM sampling. High-resolution TEM (HR-TEM) further reveals clear lattice fringes [28]. For PbSe, a lattice spacing of 0.216 nm is assigned to the (220) plane. In the PbSe/PbS sample, a lattice spacing of 0.152 nm is observed and attributed to the PbS (400) plane. This value is close to the bulk PbS spacing (0.1485 nm); the slight deviation may reflect local lattice accommodation and/or measurement uncertainty in the PbSe/PbS QDs [29].

Figure 2d shows the absorption spectra of PbSe and PbSe/PbS QDs. After PbS overcoating, an excitonic feature appears near 1530 nm and the absorption edge becomes steeper compared with PbSe, indicating reduced energetic disorder and improved surface passivation, which is plausibly associated with the PbS overcoating process [30]. Taken together, the XRD, TEM/HR-TEM, and optical absorption results support the successful introduction of PbS onto PbSe QDs and the formation of PbSe/PbS QDs. Future work will employ spatially resolved compositional analysis (e.g., STEM-EDS/EELS) to further clarify the radial composition distribution.

Figure 2e presents the nonlinear transmission of the PbSe/PbS QD-PS composite SA film, which is fitted using a two-level SA model [4]:

$$T(I)=1-\frac{\Delta T}{1+I/I_{sat}}-T_{ns}$$

Here, *I* denotes the incident intensity, Δ*T* is the modulation depth, *I_sat_* is the saturation intensity, and *T_ns_* represents nonsaturable loss. The fitting yields Δ*T* = 33%, *I_sat_* = 5.76 kW cm^−2^, and *T_ns_* = 8%, confirming a pronounced saturable-absorption response from the composite film. The inset of Figure 2e shows a photograph of the uniform freestanding PbSe/PbS QD-PS composite film used for direct fiber connector integration.

## 3. Mode-Locked Fiber Laser

**Figure 3:**
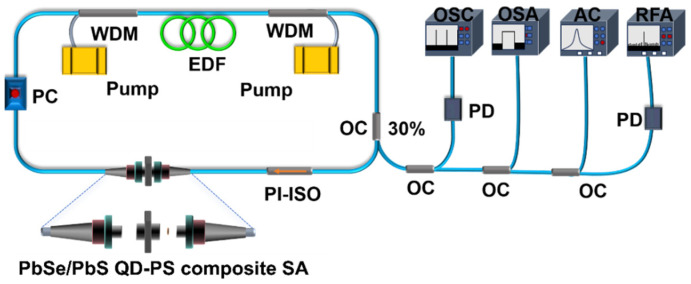
Experimental setup of the bidirectionally pumped mode-locked erbium-doped fiber laser incorporating a freestanding PbSe/PbS QD-PS composite SA.

Figure 3 shows the all-fiber ring cavity used to evaluate the freestanding PbSe/PbS QD-PS composite SA. The cavity length is 33.9 m, comprising 4.6 m erbium-doped fiber (EDF, EDFC-980-HP; absorption coefficient: 6 dB/m) and 29.3 m standard single-mode fiber (SMF-28), which yields a net anomalous group-velocity dispersion of −0.54 ps^2^. Two 980-nm laser diodes (LDs) provide bidirectional pumping via 980/1550-nm wavelength-division multiplexers (WDMs). A polarization-insensitive isolator (PI-ISO) enforces unidirectional lasing, while a polarization controller (PC) is used to access and optimize the mode-locked state. The freestanding PbSe/PbS QD-PS film is sandwiched between two FC/PC connectors, enabling connector-level integration without free-space alignment. Laser output is extracted through a 70:30 optical coupler (OC). The output characteristics are measured using an optical spectrum analyzer (OSA, Yokogawa AQ6370D, Yokogawa Electric Corporation, Tokyo, Japan), an autocorrelator (AC, APE PulseCheck SM1600, APE GmbH, Berlin, Germany), a digital oscilloscope (OSC, RIGOL DS4050, RIGOL Technologies, Inc., Beijing, China), and a radio-frequency spectrum analyzer (RFA, Rohde & Schwarz FSV30, Rohde & Schwarz GmbH & Co. KG, Munich, Munich, Germany).

**Figure 4:**
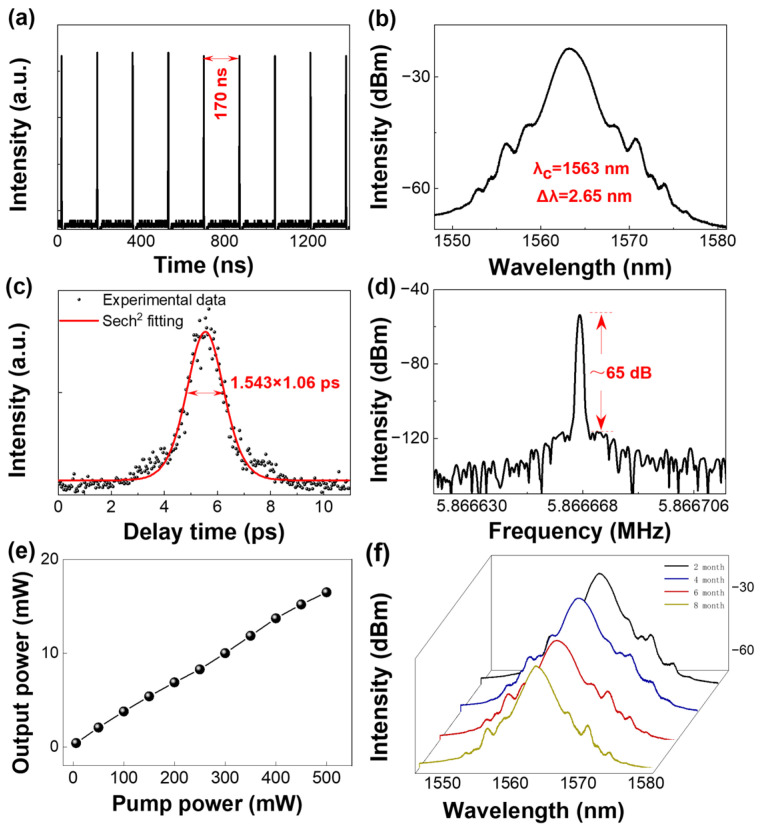
(**a**) Oscilloscope trace of the mode-locked pulse train. (**b**) Optical spectrum. (**c**) AC trace with a sech^2^ fit. (**d**) RF spectrum around the fundamental repetition frequency. (**e**) Average output power as a function of the pump power. (**f**) Optical spectra recorded during an 8-month stability test.

With the composite SA inserted, self-starting mode locking is obtained at a total pump power of 6 mW with *P_f_* = *P_b_* = 3 mW. The oscilloscope trace in Figure 4a shows a pulse-to-pulse spacing of 170 ns, corresponding to a repetition rate of ~5.86 MHz, consistent with the 33.9-m cavity length. The spectrum (Figure 4b) is centered at 1563 nm and features clear Kelly sidebands, indicating soliton operation in the anomalous-dispersion regime. From the autocorrelation trace (Figure 4c), the pulse width is 1.06 ps assuming a sech^2^ profile. Using the measured 3-dB bandwidth of 2.65 nm, the time-bandwidth product is 0.345, close to the transform-limited value of 0.315 for sech^2^ pulses, suggesting only weak residual chirp [31]. The RF spectrum (Figure 4d) presents a distinct fundamental peak at 5.86 MHz with an SNR of ~65 dB (RBW = 100 Hz), supporting stable mode-locked operation. When the bidirectional pumps are increased to *P_f_* = *P_b_* = 250 mW (total pump power of 500 mW), the average output power rises nearly linearly to 17 mW (Figure 4e), demonstrating that the SA device maintains effective operation over the tested pump range. Under 5.86 MHz repetition-rate conditions, the optical damage threshold of the PbSe/PbS QD-PS composite SA is 13.6 mJ·cm^−2^, indicating strong optical robustness. Long-term stability is assessed by tracking the spectra for 8 months; as shown in Figure 4f, the spectral envelope and bandwidth remain essentially unchanged, confirming good environmental and operational durability.

## 4. Discussion and Conclusions

Low-threshold initiation in mode-locked fiber lasers is primarily influenced by (i) the gain distribution along the active fiber and (ii) the ability of the SA to provide effective nonlinear loss modulation at low intracavity intensity. In our system, both aspects are addressed by integrating bidirectional pumping with a freestanding PbSe/PbS QD-PS composite SA. The low saturation intensity of the composite SA enables saturable absorption to take effect at a relatively low onset intensity, facilitating pulse initiation from noise without excessive attenuation of initial fluctuations. In relatively long EDF cavities, a strongly nonuniform inversion profile can increase the intracavity intensity required for self-starting. By introducing the 980-nm pump from both ends of the cavity, this nonuniformity is mitigated, resulting in a more uniform gain distribution and reducing the intracavity energy required for pulse build-up. Together, these effects enable self-starting soliton mode locking at a total pump threshold as low as 6 mW.

The observed stability and power tolerance of the SA are likely related to the PbS overcoating on PbSe and the polymer-based composite film. The PbS overcoating is expected to reduce trap-assisted losses and slow oxidation processes, which is consistent with the large modulation depth and the absence of significant spectral drift over an 8-month monitoring period under the tested conditions. In addition, the composite film shows an optical damage threshold of 13.6 mJ·cm^−2^, and near-linear output power scaling up to 17 mW was achieved in our experiment without visible degradation, indicating robust all-fiber operation at the time of measurement. Table 1 summarizes the comparative performance of mode-locked lasers incorporating the PbSe/PbS QD-PS composite SA and other reported composite SA structures. Overall, these results indicate that the PbSe/PbS QD-PS composite SA provides a favorable combination of broadband nonlinearity, low saturation intensity, thermal robustness under the tested conditions, and long-term spectral stability, supporting its potential for high-performance, long-term operation in ultrafast fiber laser systems.

From a practical perspective, the freestanding film also enables straightforward connector-level integration, making it suitable for compact ultrafast systems requiring reliable long-term operation. From a scalability and cost perspective, the PbSe/PbS QD-PS composite film is prepared via solution-phase colloidal synthesis followed by solution-based polymer composite film fabrication, which is in principle compatible with scale-up.

Environmental and health considerations associated with lead-based QDs are important. In this work, the PbSe/PbS QDs are encapsulated within a polystyrene matrix and used as a connector-integrated freestanding film, which minimizes direct exposure and reduces the likelihood of Pb release during operation.

In summary, a freestanding PbSe/PbS QD-PS composite SA is realized and implemented in a bidirectionally pumped erbium-doped fiber laser operating in the anomalous-dispersion regime. Self-starting soliton mode locking is achieved at 6 mW total pump power, producing 1.06 ps pulses with an RF SNR of ~65 dB. The SA combines low saturation intensity (5.76 kW·cm^−2^), strong modulation (33%), high damage tolerance (13.6 mJ·cm^−2^), and stable spectra over 8 months, providing a practical and scalable route toward low-threshold, durable ultrafast fiber lasers.

## Figures and Tables

**Table 1:** Comparison of composite/heterostructured SA mode-locked EDFLs.

SA	Δ*T* (%)	*P*th (mW)	*P*_out_ (mW)	SNR (dB)	Refs.
PbSe/PbS QDs	33	6	17	65	this work
PbS/CdS QDs	8.2	200	10	50	[15]
InP/ZnSeS/ZnS QDs	24.2	150	1.08	50	[16]
PbS/CdS QDs	4.95	NA	2.71	50	[32]
Te@Se	12.1	180	2.9	45	[33]
VO_2_/V_2_O_5_	23	140	NA	75	[34]
ZIF-8@ZIF-67	2.4	100	9.53	57	[35]

Δ*T* is modulation depth, *P*th is self-starting threshold, *P*_out_ is output power, SNR is signal-to-noise ratio, Refs. is references.

## Data Availability

The data is available on request from corresponding author.
